# Identifying factors associated with the uptake of prevention of mother to child HIV transmission programme in Tigray region, Ethiopia: a multilevel modeling approach

**DOI:** 10.1186/1472-6963-14-181

**Published:** 2014-04-23

**Authors:** Wondwossen Lerebo, Steven Callens, Debra Jackson, Christina Zarowsky, Marleen Temmerman

**Affiliations:** 1 School of Public Health, University of the Western Cape, Cape town, South Africa; 2 College of Health Science, Mekele University, Mekele, Ethiopia; 3 Department of Internal Medicine, Infectious Diseases and Psychosomatic Medicine University Hospital Ghent, Ghent, Belgium; 4 Department of Obstetrics and Gynecology, University Hospital Ghent and International Centre for Reproductive Health, Ghent University, Ghent, Belgium

## Abstract

**Background:**

Prevention of mother to child HIV transmission (PMTCT) remains a challenge in low and middle-income countries. Determinants of utilization occur – and often interact - at both individual and community levels, but most studies do not address how determinants interact across levels. Multilevel models allow for the importance of both groups and individuals in understanding health outcomes and provide one way to link the traditionally distinct ecological- and individual-level studies. This study examined individual and community level determinants of mother and child receiving PMTCT services in Tigray region, Ethiopia.

**Methods:**

A multistage probability sampling method was used for this 2011 cross-sectional study of 220 HIV positive post-partum women attending child immunization services at 50 health facilities in 46 districts. In view of the nested nature of the data, we used multilevel modeling methods and assessed macro level random effects.

**Results:**

Seventy nine percent of mothers and 55.7% of their children had received PMTCT services. Multivariate multilevel modeling found that mothers who delivered at a health facility were 18 times (AOR = 18.21; 95% CI 4.37,75.91) and children born at a health facility were 5 times (AOR = 4.77; 95% CI 1.21,18.83) more likely to receive PMTCT services, compared to mothers delivering at home. For every addition of one nurse per 1500 people, the likelihood of getting PMTCT services for a mother increases by 7.22 fold (AOR = 7.22; 95% CI 1.02,51.26), when other individual and community level factors were controlled simultaneously. In addition, district-level variation was low for mothers receiving PMTCT services (0.6% between districts) but higher for children (27.2% variation between districts).

**Conclusions:**

This study, using a multilevel modeling approach, was able to identify factors operating at both individual and community levels that affect mothers and children getting PMTCT services. This may allow differentiating and accentuating approaches for different settings in Ethiopia. Increasing health facility delivery and HCT coverage could increase mother-child pairs who are getting PMTCT. Reducing the distance to health facility and increasing the number of nurses and laboratory technicians are also important variables to be considered by the government.

## Background

With an estimated 1.1 million people living with HIV, Ethiopia has one of the largest populations of HIV infected people in the world [1]. The Government of Ethiopia has started integrating services such as prevention of mother to child HIV transmission (PMTCT) and HIV counseling and testing (HCT) within family planning and maternal, newborn and child health services.

Despite the overall increase in PMTCT coverage and uptake in developing countries, it remains low and unevenly distributed. Access to PMTCT services remains a challenge in the fight against HIV/AIDS and for reaching the Millennium Development Goals (MDGs) in Ethiopia. Mother to child HIV transmission (MTCT) remains high: UNAIDS estimated that almost 330,000 children were infected, and 230,000 children under fourteen years of age died of HIV in 2011 [2]. Around 90 percent of child infections occur in sub-Saharan Africa [2], and Ethiopia is not an exception.

Interventions exist that can reduce MTCT from 20% - 45% to less than 1% - 2% [3-5]. Vertical transmission of HIV in low- and middle-income countries has declined to less than 5% in the best-case scenarios after the introduction PMTCT strategies [6-8], and in wealthy countries, transmission rates are below 2% [9,10].

Although Ethiopia has made progress in the provision of services to reduce MTCT by increasing the proportion of women getting tested and knowing their results through expanding rapid testing to many PMTCT sites, national ANC coverage is only 66.3%, and coverage of skilled birth at a health institution is a mere 24.9%. HIV testing, antenatal care and skilled birth attendants influence the utilization of PMTCT [11].

Ethiopia is one of the six countries that account for 50% of under-5 child deaths worldwide, with approximately 350,000 Ethiopian children dying each year [12]. Eleven percent of child deaths result from HIV/AIDS [13]. To avert these deaths, detection of maternal infection early in pregnancy through HCT and access to antiretroviral prophylaxis is crucial. Generally, the uptake of these interventions remains low, primarily due to low ANC uptake and poor antenatal HIV testing rates [14,15].

In Ethiopia, a total of 1,023 health facilities were providing PMTCT services at the end of 2009. More than 616,763 pregnant women had at least one ANC visit in 2009, and 417,841 women underwent HCT, of whom 10,267 (2.4%) tested positive. Of all the pregnant women diagnosed with HIV, only 6,466 (63%) received antiretroviral prophylaxis (ARV/NVP) and only 5,025 infants received PMTCT prophylaxis in the same year. Of the total estimated 84,189 HIV-positive pregnant women in 2009 only 8% received ARV/NVP during childbirth [11].

Several papers have studied the determinants of getting PMTCT services. Cultural and social barriers that may prevent receipt of PMTCT services have not received adequate attention [16]. In general, challenges cited include limited screening for HIV in children, lack of affordable, simple diagnostic testing technologies, lack of human capacity, insufficient advocacy and poor understanding that ART is efficacious in children [15]. Evidence also suggests that factors operate at both the micro and macro level in getting PMTCT services. Micro-level factors include health seeking behaviour [17], adherence [18,19], home delivery [14], non-attendance of ANC [14], lack of knowledge [20], stigma [20], discrimination [20], trust in the hospital [21], while the macro level includes factors such as underlying inequities in healthcare quality [22], health services [17], health policy [17], distance and transport cost [21].

A drawback of these studies has been the use of single level analytical techniques that ignore clustering and the hierarchical structure of data for individuals living in different households, neighborhoods, cities, and provinces. Multilevel modeling can account for factors at individual and community levels simultaneously and provide a more robust understanding of the factors associated with receiving PMTCT services [23].

**Figure 1:**
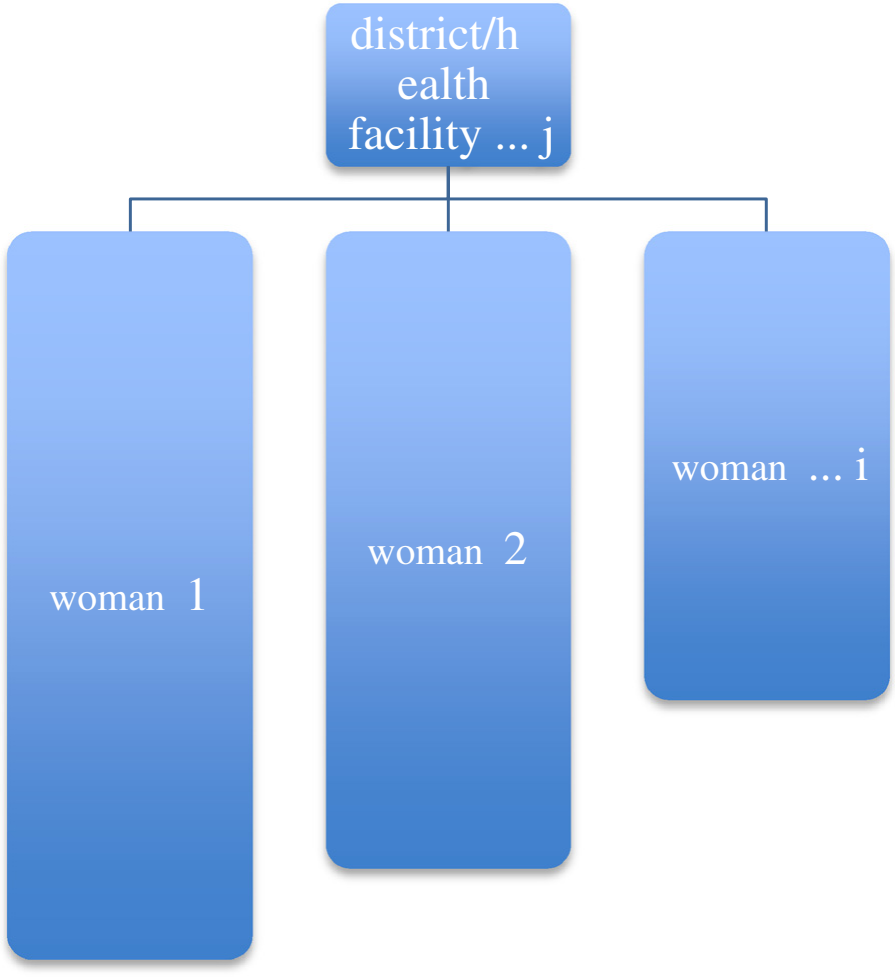
The heirarchical system diagram (women is nested in the health facility/district).

The main reason to apply multilevel modeling is that many kinds of data, including observational data collected in the human and biological sciences have a hierarchical or clustered structure [24]. Individuals interact with the social contexts to which they belong, meaning that individual persons are influenced by their social groups or contexts, and that the properties of those groups are in turn influenced by the individuals who make up that group. Generally, the individuals and the social groups are conceptualized as a hierarchical system (see Figure 1), with individuals and groups defined at separate levels of this hierarchy. Naturally, such systems can be observed at different hierarchical levels, and variables may be defined at each level [25]. Because of growing statistical technology and increasing interest in societal influences on individual health status, group level and individual level factors in regression models have prompted interest in contextual research in epidemiology [26]. The statistical issues involved in multilevel studies have been well described, and hierarchical regression analysis is becoming widely accepted as the appropriate tool for examining group level effects on individual health [27]. However, as far as we can determine, this variation in health has received little attention in public health until recently and is almost invisible in African studies on receiving PMTCT services.

By explicitly acknowledging the existence of groups, modeling group-to-group variation simultaneously with individual-to-individual variation, and including group-level properties with individual-level variables in the analyses, multilevel models allow for the importance of both groups and individuals in understanding health outcomes. They provide one way to link the traditionally distinct ecological- and individual-level studies and to overcome the limitations inherent in focusing only at one level. Like other statistical methods, multilevel analysis helps to describe, summarize, and quantify patterns present in the data [23].

**Figure 2:**
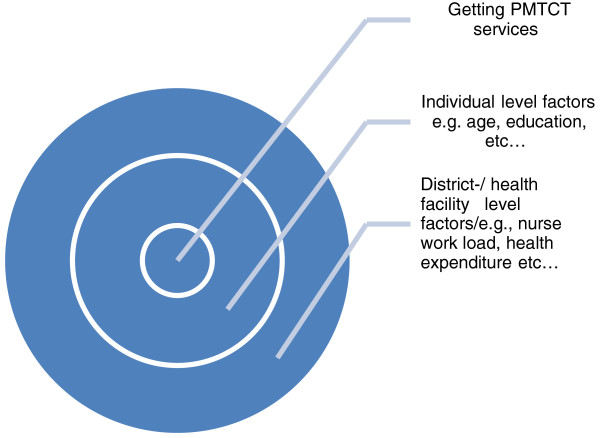
Conceptual model of macro- and micro-level factors affecting getting PMTCT services.

Although several individual characteristics have been associated with getting PMTCT services (e.g., age, education), associations with contextual characteristics, such as nurse load, PHC per population (see Figure 2), have largely been understudied. Several theoretical frameworks [28,29] have, however, stressed that the immediate environment (e.g., home or community context) may influence individuals’ health behaviour. Nevertheless, most studies on PMTCT have applied single-level analytic techniques, ignoring the social context within which individuals live [30-32]. This study explicitly examined both individual and contextual level correlates of receiving PMTCT services, by implementing a multilevel methodological approach.

The research questions that this study explored are the following: What are the individual and contextual factors that affect receipt of PMTCT services? Which determinants (community or individual level) are influential for receipt of PMTCT services inter and intra districts (woredas) in Tigray region Ethiopia?

## Methods

The economy of Ethiopia is dependent on agriculture, contributing 47% to the Gross National Product (GNP) and accounting for more than 80% of exports, as well as providing employment for 85% of the population [33]. The Tigray Region has a total population of more than 4.3 million; urban inhabitants make up 19.5% of the population [34]. The estimated population density is 86.15 people per square kilometre. The region has 1 million households, with an average of 4.4 persons to a household, with urban households having on average 3.4 and rural households 4.6 people.

Only half of the population has access to health services. The ANC coverage in Tigray region in 2009 was 73.0%, 43% of mothers utilizing ANC were tested for HIV, and of these, 3.1% tested positive [35]. Only half of HIV positive pregnant women and 38% of babies born to HIV positive mothers were given single dose nevirapine (sdNVP) or combination antiretroviral treatment (cART).

From May 05 to July 15, 2011 a health facility based cross-sectional study was conducted in Tigray, Ethiopia. The participants were selected by using a multistage sampling. Forty six districts, comprising 13 hospitals and 208 health centers were determined by the Tigray region Bureau of Health [35]. One health center from each district randomly and all available hospitals were chosen. Thirty to 36 post-partum women were purposively selected from each health facility. The managers of all of the selected health facilities and districts were also interviewed.

All participants were interviewed face to face by a trained nurse using structured questionnaire to collect information on demographic, socio-economic characteristics and on women’s maternal healthcare, for instance, prenatal care, delivery and postnatal care related to PMTCT. Informed consent was obtained from each participant at the start of the survey. The study was approved by the ethics committee of University of the Western Cape, and Tigray region Health Bureau.

“Getting PMTCT service” during the last pregnancy and delivery was used as the principal outcome indicator in the analysis of the demographic and socio-economic determinants at the individual and community level. This indicator, coded 1 for “yes” or 0 for “no”, was defined in accordance with the Ministry of Health PMTCT guidelines as follows: the mother was asked if she received antiretroviral medication, before, after or/and at the time of labour depending on her clinical stage, and whether the child received prophylaxis at the time of birth or/and after birth depending on the mother’s clinical stage.

A two level logistic regression model was used to assess the explanatory effects of the independent variables on getting PMTCT services by considering the hierarchical structure of the study sample. The first level represents the individual and the second level is the district/community. The community level covariate was the geographical demarcation of the districts.

Individual level covariates comprised age group (≤24, 25–34, or ≥35) years; education (categorized as none, primary, or secondary/higher); number of pregnancy (≤2 or ≥3); place of delivery (home or health facility); ever stigmatized (yes or no); ever discriminated (yes or no); planned pregnancy (yes or no); any CD_4_ count done (yes or no); time of HIV status knowledge (before being pregnant, during pregnancy, at the time of delivery, or after giving birth); HIV status disclosure to the spouse (yes or no); who attended the birth of child (traditional birth attended, doctor, or nurse/mid-wife/other); and socio-economic status (SES) quintile (1^st^ quintile (poorest), 2^nd^ quintile, 3^rd^ quintile, 4^th^ quintile, or 5^th^ quintile (wealthiest)). The quintile combined information on a set of household assets and living conditions: the household income, employment status, main source of water, type of toilet, main fuel used for cooking, and main material the house built.

Data on the community level variables included in the model were obtained from the questionnaires for mothers health facility and district managers and complemented by official data from secondary source [35]. Place of residence grouped as urban, rural; proportion of women with no education in the district grouped as <30%, 30-50%, >50%; proximity grouped as <1 km, 1-5 kms, >5 kms; nurse workload defined as ≤1500, >1500 people per nurse; proportion of poor and poorest household in the district grouped as <30%, 30-60%, >60%; people per health worker defined as ≤500, >500; people per health facility defined as ≤25000, >25000; lab technician workload defined as ≤3100, >3100 people; and people per HCT site grouped as ≤25000, >25000.

The community level random effects were estimated, using xtmelogit function, at a 2-level multilevel model as shown:

(1) $$\log\left(\frac{\pi_{\mathrm{ij}}}{1-\pi_{\mathrm{ij}}}\right)=\beta_{0}+\beta_{1}X_{\mathrm{ij}}+u_{0j}$$

With β_0_ as the intercept and the slope β_1,_ defined as the expected change in getting PMTCT service. A set of intercepts was estimated for the community level, where ^π^ij is the probability of utilizing HCT for a pregnant woman i, in a district j, and β0j is a parameter associated with the fixed part of the model. Therefore, for every one unit increase in X (a set of predictor variables) there is a corresponding effect on the probability mother or child getting PMTCT service. By assuming that each community has a different intercept β_0j_ and a different slope β_1j_ the clustered data structure and the within and between community variations is now taken into account. To capture the extent by which choice of different option of getting PMTCT service, which are contrast specific, varies randomly at the individual level, the results of random effects (measures of variation) are presented as variance partition coefficient (VPC):

(2) $$\mathrm{vpc}=\frac{\sigma_{u0}^{2}}{\sigma_{\mathrm{uo}}^{2}+\frac{\pi^{2}}{3}}$$

Where, π^2^/3 denotes the variance between mother or child from the same district (individual level) and *σ*2*u*0 is the variance between districts (community level variance).

Data analyses were conducted using Stata 11 (Stata Corp. Inc., TX, USA). The statistical significance of the explanatory variables was estimated using Wald statistics, with all results at <5% alpha level considered significant. The results of the fixed (measure of association) effects were presented as odds ratio (OR) at their 95% confidence intervals (95% CIs).

As this study used several explanatory variables that might be correlated to each other (such as mother’s education, father’s education and household wealth index), the multicollinearity assessment was conducted using the means of variance inflation factors and it is small (1.22) indicated the absence of any significant collinearity between explanatory variables in the regression model.

## Results

Among the 220 HIV positive mothers, 95.4% have provided information about PMTCT. Of these, 79% of mothers and 55.7% of children reported receiving PMTCT services (Table 1).

**Table 1:** Background individual and community-level characteristics of post-partum women who were attending health facility for the child immunization and mother and child PMTCT service in Tigray region Ethiopia

**Variables**	**n**	**Mother PMTCT%**	**Child PMTCT%**
Age group (years)			
≤24	35	71,4	68,6
25-34	140	81,4	50,7
≥34	33	75,8	63,6
Mother education			
No education	90	77,8	61,1
Primary	81	76,5	53,1
Secondary/Higher	38	89,5	47,4
Father education			
No education	56	75,0	57,1
Primary	81	82,7	61,7
Secondary/Higher	66	80,3	47,0
Religion			
Orthodox	191	78,0	55,5
Non Orthodox	19	89,5	57,9
Household size			
≤3	153	81,0	59,5
>3	23	69,6	34,8
Number of pregnancy			
≤2 times	102	78,4	55,9
>2 times	93	78,5	54,8
Age of first pregnancy			
≤25 years	167	78,4	55,1
>25 years	38	84,2	60,5
Utilized ANC			
No	34	70,6	41,2
Yes	175	80,6	58,9
Where was the child born?			
Home	27	33,3	25,9
Health facility	182	86,3	60,4
Have you ever stigmatized?			
Yes	113	80,5	54,9
No	97	77,3	56,7
Have you ever discriminated?			
Yes	81	77,8	59,3
No	126	81,8	54,8
Confidentiality with health workers			
No	128	82,8	55,5
Yes	81	74,1	56,8
Knowledge level of HIV			
Poor	98	78,6	49,0
Good	112	79,5	61,6
Planned pregnancy			
No	72	81,9	65,3
Yes	132	78,0	50,8
Discussed the best way to feed with anyone			
No	36	72,2	55,6
Yes	170	80,6	56,5
Have you had CD4 count?			
Yes	183	84,7	59,0
No	25	40,0	36,0
Father of child tested for HIV			
No/Don’t Know	69	75,4	60,9
Yes	139	80,6	52,5
Get encouragement from husband to HCT			
No	72	81,9	68,1
Yes	135	77,8	48,9
Time you knew HIV status			
Before being pregnant	109	79,8	45,9
While I was pregnant	83	80,7	69,9
At the time of delivery	13	92,3	69,2
After giving birth	5	0,0	0,0
Have you told your HIV status to anyone?			
Yes	55	70,9	61,5
No	153	81,7	50,9
Know site that gives HCT			
No	20	80,0	50,0
Yes	189	79,4	56,6
Who attended the birth of child?			
Traditional birth attendant	28	32,1	25,0
Doctor	26	80,8	61,5
Nurse/Mid-wife/Other	155	87,7	60,7
SES quintile			
1st quintile(poorest)	47	68,1	48,9
2nd quintile	59	78,0	59,3
3rd quintile	56	82,1	55,4
4th quintile	27	88,9	70,4
5th quintile(richest)	21	85,7	42,9
Place of residence			
Urban	72	85,4	57,7
Rural	137	66,7	51,4
Proximity			
<1 km	62	88,7	54,8
1-5 kms	42	85,7	69,1
>5 kms	37	59,5	48,6
Number of people per Nurse			
≤1500	86	84,9	47,7
>1500	124	75,0	61,3
Number of people per health workers			
≤500	71	85,9	47,9
>500	139	75,5	59,7
Number of people per health facility			
≤25000	170	78,2	55,3
>25000	40	82,5	57,5
Number of people per laboratory technician			
≤3100	112	85,7	56,2
>3100	98	71,4	55,1
Proportion of poorest and poor household			
<30%	73	78,1	58,9
30-60%	78	75,6	57,7
>60%	59	84,8	49,2
Proportion of women with no education			
<30%	104	77,9	54,8
30-50%	83	77,1	59,0
>50%	23	91,3	47,8
Ever HIV test kit run out of stock?			
No	40	67,5	40,0
Yes	170	81,8	59,4
Ever ART run out of stock?			
No	145	77,9	55,2
Yes	65	81,5	56,9
Test syphilis during pregnancy?			
No	21	85,7	66,7
Yes	189	78,3	54,5
The number of HCT site in the district			
<3	78	85,9	47,4
3-5	108	74,1	60,2
>5	24	79,2	62,5
The number of PMTCT sites in the district			
None	8	75,0	37,5
≤2	171	80,1	56,7
>2	31	74,2	54,8

### Multilevel analysis

#### Crude multilevel modeling result

In crude multilevel modeling the odds of getting PMTCT service were 14 times (OR = 13.99; 95% CI 4.94,39.60) higher for a mother delivering at a health facility, and a child born at a health facility was 7 times (OR = 6.93; 95% CI 2.27,21.14) more likely to get PMTCT services than a mother delivering and child born at home, respectively. The bivariate multilevel modeling showed that having a CD4 count significantly increased the odds of receiving PMTCT services for both mother (OR = 8.30; 95% CI 3.39,20.33) and child (OR = 3.11; 95% CI 1.17,8.25), compared to who did not receive a CD4 count. Contrary to our expectation, planned pregnancy (OR = 0.54; 95% CI 0.28,1.05) and getting encouragement from husband to access HCT (OR = 0.42; 95% CI 0.21,0.83) decreased the chance of getting PMTCT services for a child. Per our expectation a mother coming from an urban area was 3.2 times (OR = 3.19; 95% CI 1.46,6.97) more likely to receive PMTCT services compared to her rural counterparts. Proximity to health facility and laboratory technician workload were associated with getting PMTCT services for women (Table 2).

**Table 2:** Results of the multilevel analysis of post-partum women who were attending health facility for the child immunization and mother and child PMTCT services in Tigray region Ethiopia

**Variables**	**PMTCT Mother**		**PMTCT Child**	
	**Crude OR(CI)**	**Adjusted OR(CI)**	**Crude OR(CI)**	**Adjusted OR(CI)**
Household size				
≤3			2.85(1.088-7.456)	3.03(0.963-9.508)
>3			1	1
Where was the child born?				
Home	1	1	1	1
Health facility	13.99(4.940-39.595)	18.21(4.369-75.908)	6.93(2.274-21.142	4.77(1.209-18.834)
Planned pregnancy				
No			1	1
Yes			0.54(0.277-1.054)	0.39(0.157-0.960)
Have you had CD4 count?				
Yes	8.30(3.391-20.335)		3.11(1.170-8.250)	3.47(1.052-11.431)
No	1		1	1
Get encouragement from husband to HCT				
No			1	1
Yes			0.42(0.210-0.834)	0.37(0.156-0.892)
Time you knew HIV status				
Before being pregnant	1		1	
While I was pregnant	1.06(0.514-2.192)		3.50(1.675-7.293)	
At the time of delivery	3.04(0.373-24.804)		3.68(0.904-14.993)	
After giving birth	0.00(0.000-8)		0.00(0.000-8)	
Who attended the birth of child?				
Traditional birth attendant	1		1	
Doctor	9.06(2.471-33.233)		7.16(1.814-28.300)	
Nurse/Mid-wife/Other	15.55(5.718-42.308)		7.21(2.403-21.651)	
Place of residence				
Urban	3.19(1.461-6.968)			
Rural	1			
Proximity				
<1 km	5.36(1.923-14.921)	4.57(1.206-17.345)		
1-5 kms	4.09(1.382-12.109)	2.36(0.666-8.390)		
>5 kms	1	1		
Number of people per Nurse				
≤1500	1.89(0.894-3.991)	7.22(1.016-51.265)	0.58(0.287-1.176)	0.41(0.175-0.976)
>1500	1	1	1	1
Number of people per health workers				
≤500	1.98(0.912-4.277)			
>500	1			
Number of people per laboratory technician				
≤3100	2.40(1.207-4.771)	9.27(1.555-55.238)		
>3100	1	1		
Ever HIV test kit run out of stock?				
No	1	1	1	
Yes	2.38(0.948-5.974)	3.48(0.813-14.920)	2.83(1.062-7.524)	

#### Mother adjusted multilevel modeling result

Multivariate multilevel modeling (adjusted) (Table 2) showed that, after controlling for the variables that were significant in bivariate multilevel modeling. A mother who was delivering at health facility had almost a 18 fold (AOR = 18.21; 95% CI 4.37,75.91) higher chance to receive PMTCT services than a mother who gave birth at home. More of the community level variables were significantly associated with mother getting PMTCT services than the child. A mother who was living within the <1 km radius proximity to health facility had higher odds (AOR = 4.57; 1.21,17.34) of getting PMTCT services than a mother who was living within >5 kms radius. For every addition of one nurse per 1500 people, the likelihood of getting PMTCT services for a mother increases by 7.22 fold (AOR = 7.22; 95% CI 1.02,51.26), when other individual and community level factors were controlled simultaneously. In addition to this, adding one laboratory technician for every 3100 people, improves the odds of getting PMTCT services for a mother by 9.27 fold (AOR = 9.27; 95% CI 1.55,55.24) (Table 2).

#### Child adjusted multilevel modeling result

The results in Table 2 showed that, a child born at a health facility had almost 7 times (OR = 6.93; 95% CI 2.27,21.14) and almost 5 times (AOR = 4.77; 95% CI 1.21,18.83) higher chance to get PMTCT services compared to a child born at home, before and after controlling for other variables, respectively. A child born to a mother who had a CD4 count was 3.5 times (AOR = 3.47; 95% CI 1.05,11.43) more likely to get PMTCT services than the child born to a mother whose CD4 count was not done, controlling for the other variables. Another individual level factor significantly (though negatively) associated with child getting PMTCT service even after controlling for other variables was getting encouragement from husband to have HCT; a child born to a mother who got this encouragement was 63% less (AOR = 0.37; 95% CI 0.16,0.89) likely to receive PMTCT services than a child born to a mother who did not get the encouragement. Among the community level variables “ever HIV test kit run out of stock” was significantly associated with child getting PMTCT services, even though, the significance disappeared when it was adjusted for the other variables. Nurse work load was the only community level variable that was significantly associated with child getting PMTCT service when other variables were controlled; but against our expectation adding one nurse for every 1500 people decrease the likelihood of child getting PMTCT service by 59% (AOR = 0.41; 95% CI 0.18,0.98).

#### Random effect result

The community level VPC was 4.6%, showing there was a slight difference in getting PMTCT services for mothers at the community level; however, this slight difference disappeared (VPC = 0.6%) when different variables were controlled for. This confirmed minimal difference in getting PMTCT services at the community level (Table 3).

**Table 3:** Random effect results of the post-partum women who were attending health facility for the child immunization and mother PMTCT services in Tigray region Ethiopia

**Random effect**	**Model 1**	**Model 2**	**Model 3**	**Model 4**
Intercept	1.37(0.203)	−1.73(1.518)	−0.50(1.554)	−0.94(1.124)
Community-level variance (SE)	0.16(0.370)	0.07(0.741)	0.00(0.000)	0.02(0.789)
VPC (%)	4.6	2.1	0.0	0.6
Proportional change in community level variance	Reference	56.2	100	87.5
**Model fit statistics**				
DIC(−2log likelihood)	215.3306	139.0594	115.3768	98.0654

Model 1 Null model.

Model 2 Only individual level explanatory variables included in the model.

Model 3 Only community level explanatory variables included in the model.

Model 4 Full model.

The child multivariate multilevel modeling showed that the community level VPC was 12.5% for the null model. When the model is controlled for the variables that were significant in crude multilevel modeling, the VPC increased to 27.2% (Table 4).

**Table 4:** Random effect results of the post-partum women who were attending health facility for the child immunization and child PMTCT services in Tigray region Ethiopia

**Random effect**	**Model 1**	**Model 2**	**Model 3**	**Model 4**
Intercept	0.27(0.190)	−6.14(2.325)	0.99(1.345)	−2.32(1.242)
Community-level variance (SE)	0.47(0.401)*	0.70(0.650)*	0.27(0.379)	1.23(1.086)*
VPC (%)	12.5	17.5	7.6	27.2
Proportional change in community level variance	Reference	48.9	42.6	61.8
**Model fit statistics**				
DIC(−2log likelihood)	285.2762	165.2646	178.6708	116.3478

*significant at p-value < 0.05.

Model 1 Null model.

Model 2 Only individual level explanatory variables included in the model.

Model 3 Only community level explanatory variables included in the model.

Model 4 Full model.

## Discussion

The implementation of strategies to eliminate MTCT remains a major challenge in developing countries [36-39]. To our knowledge, this study constitutes the first multilevel analysis to explore factors associated with receiving PMTCT services in Ethiopia. The proportion of mothers getting PMTCT services (79%) in our study were comparatively higher than the findings reported from Tigray Bureau of Health in 2010 (47.9%) [35], Addis Ababa in 2009 (53.7%) [40], Oromia in 2008 (35%) [19], Ethiopia in 2009 (18%) [18], the Eastern and Southern Africa region in 2011 (64%) [37], and India (60%) [39]. The proportion of children getting PMTCT services (55.7%) was higher than reported from Tigray Bureau of Health in 2010 (26.9%) [35], Addis Ababa in 2009 (40.7%) [38]. Oromia in 2008 (29%) [19], Ethiopia in 2009 (15%) [18]. This might be due to the fact that the study was limited to health facility attending mothers and ignored the mothers who did not patronize the child immunization in the participating facilities.

This study revealed that the mothers delivering at a health facility and children born at a health facility have increased chances of getting PMTCT services in Ethiopia. This finding is consistent with the studies conducted in Ethiopia [41], and elsewhere [42,43]. PMTCT services in Ethiopia are accessed only in health facilities. Children born elsewhere have less chance to get PMTCT services if their parents did not bring them to a health facility. This underlines the importance of health facility based delivery in Ethiopia to decrease significantly MTCT and to achieve MDG goal 4. However, 16% mothers and almost 40% children did not get PMTCT services at the delivery in spite of having given birth at a health facility. This puts the mothers and children at increased risk of MTCT, and implies a health system failure; especially of the birth attendant in the health facility. Therefore, continued training on the PMTCT guidelines for birth attendants and improved supervision may reduce the number of missed opportunities. At the individual level, mothers having a CD4 count were more likely to receive PMTCT services as well as their children, but when adjusted for the other variables the significant association for the mother disappeared. In contrast to our expectation getting encouragement from husband to HCT appeared to decrease the probability of child getting PMTCT service. The counterintuitive results here, as well as on the negative impact of improved staffing levels on the likelihood of a child receiving PMTCT, need further examination. We suggest that they might reflect the weak integration of post-delivery aspects of PMTCT into both programmes and parents’ expectations, if supportive husbands and better PMTCT staffing also indicate a stronger focus on pregnant women rather than on the HIV risk of children. The data in our study cannot answer – but do raise – possible important questions for further research.

At the contextual level, the current study found facility proximity, nurse work load, and laboratory technician work load were significantly associated with mothers getting PMTCT services; even when adjusted for the other variables. Our study finding showed that when the health facility was nearer to the place where the mother lives, the probability of getting PMTCT services increase dramatically. In addition to this our study revealed that, increasing the number of nurses and laboratory technicians to a given community increases significantly the likelihood of mothers getting PMTCT services. This finding is consistent with the other studies conducted previously [9,16-21]. This might be due to proximity and large number of health workers; they know easily the status of the mother and are able give all the available services, including PMTCT when she come to deliver. However, for the children all except nurse load community level variables were not significantly associated in getting PMTCT services.

The current study showed that random effects of the contextual level were significant in mothers receiving PMTCT services. The finding implies that unmeasured factors at the contextual level determine receiving PMTCT services of mothers beyond individual level was 4.6% in null model and 0.6% in full model. This shows that the contextual level effect was very small and a mother receiving PMTCT services were mostly dictated by the individual level variables. Although in children, the random effects showed great difference at the contextual level, it did not reach statistical significance.

The current study has several limitations. Causality could not be inferred, as it has a cross-sectional design. Due to the sensitive nature and interest to get enough number of participant on the study, the sample of HIV infected mothers were not selected randomly. The study was health facility based, therefore mothers who did not come for the child immunization to get services in the participating health facilities were excluded. Another limitation might be a recall bias, as the mothers were interviewed about events that occurred many months back. Furthermore internal validity could be affected by the different data collectors. To minimize this risk, data collectors were all midwifes well acquainted with the issue under study and were given training. Even though the purposes of the study have been explained to reduce social desirability bias, it might have been introduced due to the self-report. Additionally, defining districts based on the administratively defined boundaries might misclassify individuals into an inappropriate social and cultural boundary. This could generate information biases and reduce the validity of analysis.

## Conclusions

This study sheds light on the factors that determine mother and child receiving PMTCT services, operating at individual and community levels. The multilevel modeling approach allows identifying several factors at individual (for instance, place of delivery, having CD4 count) and community level (for example proximity, nurse work load) hindering the application of PMTCT services simultaneously. This may allow differentiating and accentuating approaches for different settings in Ethiopia. The government should therefore focus on increasing health facility delivery and HCT coverage to increase mother-child pairs who are getting PMTCT service. Reducing the distance to health facility, increasing the number of nurses and laboratory technicians are also important variables to be considered by the government.

## Competing interests

The authors declare that they have no competing interests.

## Authors’ contributions

WL, DJ, SC have made substantial contributions to conception, design, analysis and interpretation of data; WL, DJ, SC, CZ, MT involved in drafting the manuscript, revising it critically for important intellectual content; and all have given final approval of the version to be published.

## Pre-publication history

The pre-publication history for this paper can be accessed here:

http://www.biomedcentral.com/1472-6963/14/181/prepub
